# Supplementary material for: Resolving the full spectrum of human genome variation using Linked-Reads
Source: Genome Res. 2019 Apr;29(4):635–45. doi: 10.1101/gr.234443.118 (PMC6442396; doi:10.1101/gr.234443.118)
Supplement: Supplemental Material [file supp_gr.234443.118_Supplemental_longranger-2.2.zip › longranger-2.2.tar.bz2!/lr2.2-public/lib/stan/cmdstan/stan/doc/api/html/functions_w.html]

Stan: Class Members


|  |  |
| --- | --- |
|  | Stan  2.5.0  probability, sampling & optimization |


- Main Page
- Related Pages
- Namespaces
- Classes
- Files

- Class List
- Class Index
- Class Hierarchy
- Class Members

- All
- Functions
- Variables
- Typedefs
- Enumerator
- Related Functions

- \_
- a
- b
- c
- d
- e
- f
- g
- h
- i
- j
- k
- l
- m
- n
- o
- p
- q
- r
- s
- t
- u
- v
- w
- x
- y
- z
- ~


All Classes Namespaces Files Functions Variables Typedefs Enumerations Enumerator Friends Macros Pages

Here is a list of all class members with links to the classes they belong to:

### - w -

- warmup
  : stan::io::stan\_csv\_timing
  , stan::mcmc::chains< RNG >
- welford\_covar\_estimator()
  : stan::prob::welford\_covar\_estimator
- welford\_var\_estimator()
  : stan::prob::welford\_var\_estimator
- while\_statement()
  : stan::gm::while\_statement
- while\_statement\_r
  : stan::gm::statement\_grammar< Iterator >
- whitespace
  : stan::gm::whitespace\_grammar< Iterator >
- whitespace\_grammar()
  : stan::gm::whitespace\_grammar< Iterator >
- windowed\_adaptation()
  : stan::mcmc::windowed\_adaptation
- write()
  : stan::io::csv\_writer
  , stan::io::dump\_writer
- write\_adapt\_finish()
  : stan::io::mcmc\_writer< M, SampleRecorder, DiagnosticRecorder, MessageRecorder >
- write\_array()
  : stan::gm::write\_array\_vars\_visgen
  , stan::gm::write\_csv\_vars\_visgen
- write\_array\_vars\_visgen()
  : stan::gm::write\_array\_vars\_visgen
- write\_array\_visgen()
  : stan::gm::write\_array\_visgen
- write\_col\_major()
  : stan::io::csv\_writer
- write\_csv\_header\_visgen()
  : stan::gm::write\_csv\_header\_visgen
- write\_csv\_vars\_visgen()
  : stan::gm::write\_csv\_vars\_visgen
- write\_csv\_visgen()
  : stan::gm::write\_csv\_visgen
- write\_diagnostic\_names()
  : stan::io::mcmc\_writer< M, SampleRecorder, DiagnosticRecorder, MessageRecorder >
- write\_diagnostic\_params()
  : stan::io::mcmc\_writer< M, SampleRecorder, DiagnosticRecorder, MessageRecorder >
- write\_dims\_visgen()
  : stan::gm::write\_dims\_visgen
- write\_error\_msg\_()
  : stan::mcmc::base\_hamiltonian< M, P, BaseRNG >
- write\_metric()
  : stan::mcmc::dense\_e\_point
  , stan::mcmc::diag\_e\_point
  , stan::mcmc::ps\_point
- write\_param\_names\_visgen()
  : stan::gm::write\_param\_names\_visgen
- write\_row\_major()
  : stan::io::csv\_writer
- write\_sample\_names()
  : stan::io::mcmc\_writer< M, SampleRecorder, DiagnosticRecorder, MessageRecorder >
- write\_sample\_params()
  : stan::io::mcmc\_writer< M, SampleRecorder, DiagnosticRecorder, MessageRecorder >
- write\_sampler\_param\_names()
  : stan::mcmc::base\_mcmc
  , stan::mcmc::base\_nuts< M, P, H, I, BaseRNG >
  , stan::mcmc::base\_static\_hmc< M, P, H, I, BaseRNG >
- write\_sampler\_params()
  : stan::mcmc::base\_mcmc
  , stan::mcmc::base\_nuts< M, P, H, I, BaseRNG >
  , stan::mcmc::base\_static\_hmc< M, P, H, I, BaseRNG >
- write\_sampler\_state()
  : stan::mcmc::base\_hmc< M, P, H, I, BaseRNG >
  , stan::mcmc::base\_mcmc
- write\_timing()
  : stan::io::mcmc\_writer< M, SampleRecorder, DiagnosticRecorder, MessageRecorder >
- writer()
  : stan::io::writer< T >


---

[ Stan Home Page ]

*© 2011–2014,
Stan Development Team.*
